# Unique Bioactive Secondary Metabolites of Ferns and Their Enhancement by Abiotic Stress: Medicinal Potential and Future Perspectives

**DOI:** 10.3390/molecules31122029

**Published:** 2026-06-10

**Authors:** Kanchan Soneji, Antoni Szumny, Katarzyna Wróblewska

**Affiliations:** 1 Department of Horticulture, Wrocław University of Environmental and Life Sciences, 50-375 Wrocław, Poland; 2 Department of Food Chemistry and Biocatalysis, Wrocław University of Environmental and Life Sciences, 50-375 Wrocław, Poland; antoni.szumny@upwr.edu.pl

**Keywords:** fern, bioactive compounds, traditional medicine, unique secondary metabolites, abiotic stress

## Abstract

Ferns represent an evolutionarily distinct group of vascular plants and constitute an underexplored source of structurally diverse secondary metabolites with potential medicinal value. Several fern-derived compounds, including sesquiterpenes, triterpenes, flavonoids, phloroglucinol derivatives, lactones, and glycosides, have been associated with antibacterial, antidiabetic, analgesic, anticancer, hepatoprotective, neuroprotective, and other biological activities. However, despite their biochemical uniqueness and long-standing use in traditional medicine, ferns remain less extensively investigated than angiosperms as sources of bioactive compounds. In addition to their natural phytochemical diversity, the production of secondary metabolites in ferns may be influenced by abiotic stressors, such as light quality and intensity, temperature, salinity, drought, water availability, and mineral nutrition. Available studies indicate that selected abiotic stress conditions can enhance the accumulation of phenolic acids, flavonoids, polyphenols, carotenoids, and related compounds in several fern families, including Aspleniaceae, Athyriaceae, Dryopteridaceae, Onocleaceae, and Thelypteridaceae. Nevertheless, information on stress-induced modulation of metabolites that are unique or highly characteristic of ferns, particularly terpenes, terpene glycosides, and specific flavonoid derivatives, remains limited. This review summarizes the current knowledge on unique secondary metabolites in ferns, their reported medicinal properties, and the potential use of abiotic stress as an elicitation strategy to enhance their production. Overall, the review highlights ferns as promising but still insufficiently explored reservoirs of bioactive metabolites and identifies key directions for future phytochemical, pharmacological, and cultivation-based research.

## 1. Introduction

Secondary metabolites act as defense compounds during stress. Plants require specific quantities of water, temperature, sunlight, minerals, and other factors for growth and development. Any deviation from optimal conditions can result in plant stress. Consequently, stress is characterized by a plant’s inability to reach its full growth and developmental potential due to various environmental factors, a phenomenon that is particularly significant in the context of climate change [1,2]. The inability to adapt to adverse conditions may have detrimental effects on plants. To address these challenges, plants have devised intricate strategies to evade or withstand stressors. The activation of signaling cascades induces changes, demonstrating the protective and adaptive responses that enable plants to survive stress. Some of the significant intracellular modifications include alterations in membrane fluidity, cellular homeostasis, production of reactive oxygen species, and an increase in the abundance of secondary metabolites [3]. Increased concentrations of secondary metabolites, namely the total phenolic content in *Brassica juncea* in response to UV light, tea polyphenols in *Camellia sinensis* in response to elevated CO_2_ levels, and triterpenoids in *Panax quinquefolius* in response to high temperature, have been observed [4,5,6]. Moreover, pharmacological studies of phytochemicals obtained from higher plants have been extensively conducted [7,8].

Ferns, or Pteropsida, encompass approximately 12,484 species, with the largest family, Dryopteridaceae, comprising 2375 species, followed by Polypodiaceae with 1787 species [9]. Globally, ferns are prominent along a latitudinal gradient, with the most species-rich regions located in the tropics near the equator and declining populations observed toward the poles [10]. India hosts a diverse array of 1200 pteridophyte species, encompassing 70 distinct families and comprising 192 genera [11], whereas 1689 species can be found in Colombia [10]. Within the plant kingdom, ferns serve as a crucial phylogenetic linkage between primitives and angiosperms. Notable differences in metabolic and biochemical mechanisms between ferns and higher plant groups exist, although the underlying reasons and implications of these differences remain unclear [12]. One of these is the lower transpiration rate in ferns. Anatomically, this may be attributed to lower sap flow, xylem structure and connectivity, and low vein density, which contribute to reduced stomatal and leaf hydraulic conductance [12,13,14,15]. Metabolomic analyses conducted by Cândido-Sobrinho et al. [16] revealed that angiosperms prioritize primary metabolism, while ferns focus on secondary metabolism [16]. Angiosperms, with higher stomatal density and conductance, allow more CO_2_ intake, boosting photosynthesis and growth through primary metabolites. Their rapid stomatal responses suggest the accumulation of primary metabolites as a crucial criterion to modulate stomatal dynamics [17]. Conversely, ferns exhibit lower photosynthetic rates and stomatal conductance but greater water-use efficiency and a higher ratio of assimilated CO_2_ directed to secondary metabolite production, which are linked to better stress tolerance. Furthermore, stomatal response in ferns is mediated by changes in leaf water status, while in higher plants it is regulated by abscisic acid [18]. Ferns differ from higher plants in metabolic pathways and structural organization, and due to their distinct evolutionary lineage, they produce unique secondary metabolites that contribute to their chemical diversity, some of which are absent in other plant species [7]. Their physiological features, along with the profound production of secondary metabolites, account for high fern resilience to abiotic stress factors, such as drought [19,20].

Phylogenetic analyses using complete genome sequences to trace the evolution of the anthocyanin (a flavonoid that plays an important role in combating stress and also acts as a pigment) biosynthetic pathway and its presence in early land plants evidenced that the pathway was complete only in the most recent common ancestor of seed plants, with many downstream enzyme orthologs missing in seedless plants, including ferns [21]. Moreover, ferns have smaller acetyl and methyl transferase gene families relative to angiosperms, according to a comparative analysis of cell wall-related genes [22]. These changes at the molecular level can account for the different compositions of secondary metabolites in ferns. Moreover, recent studies have reported the exclusive presence of certain secondary metabolites in ferns.

Recent studies focusing on intensifying metabolite production have mainly focused on higher plants. Numerous reviews focusing on the role of elicitors in enhancing secondary metabolite production in higher plants are available. Espinosa-Leal et al. [23] provided a comprehensive summary of the abiotic stress factors influencing the synthesis of certain plant secondary metabolites in vitro. Similarly, Jan et al. [24] discussed the use of abiotic and biotic elicitors in culture systems and their augmentative effect on the synthesis of secondary metabolites. Yeshi et al. [8] scrutinized the impact of various abiotic stressors on the biosynthesis of bioactive phytochemicals in plant systems, emphasizing their potential role in the discovery of therapeutic drugs. The reports are endless in the case of angiosperms; however, studies on the response of ferns to stress are scarce, let alone studies on its pharmacological effects. Therefore, this review is by far the first one that aims to summarize secondary metabolites unique to ferns and the studies on the possibility of intensifying secondary metabolite production in ferns by imposing temperature, salt, drought, and mineral stresses on these plants, as well as to highlight fern biochemical diversity and outline perspectives for future fern research.

## 2. Literature Search and Selection

A comprehensive literature search was conducted to identify relevant studies on secondary metabolites unique to ferns and the effects of abiotic stress on their production. The search was performed using the electronic databases Reaxys, SciFinder, PubMed, Scopus, Web of Science, ScienceDirect, RDiscovery, and Google Scholar. Also, patent databases such as Google patent, Espacenet, and PATENTSCOPE were searched. The following combinations of keywords were used: (“fern” OR “pteridophyte” OR “Pteridophyta”) AND (“secondary metabolite*” OR “bioactive compound*” OR “phytochemical*” OR “terpene*” OR “flavonoid*” OR “phloroglucinol*” OR “pterosin*”) AND (“unique” OR “exclusive” OR “specific”); as well as (“fern” OR “pteridophyte”) AND (“abiotic stress” OR “light stress” OR “temperature stress” OR “drought” OR “salinity” OR “salt stress” OR “nutrient stress” OR “elicitor*” OR “elicitation”).

Additional manual searches were performed by screening the reference lists of relevant review articles and key papers. The search was not limited by publication date and included all peer-reviewed articles published in English up to May 2025. Only original research articles and reviews reporting the chemical structure, biological activity, or stress-induced changes of secondary metabolites in ferns (Pteridophyta) were included.

In total, 155 articles were selected after careful screening for relevance and scientific quality. This literature forms the basis of the present review.

## 3. Secondary Metabolites in Ferns

Secondary metabolites are becoming increasingly significant in diverse sectors, including the pharmaceutical, cosmetic, and food industries, highlighting the need for dependable and effective sources of these compounds. Ferns have long been vibrant reservoirs for secondary metabolites, and pteridophytes of the family Marattiaceae contain flavonoids (apigenin, luteolin, violanthin, isoviolanthin, and muxiangrine III). Ferns in the families Pteridaceae, Adiantaceae, Aspleniaceae, and Davalliaceae contain phenolics (rutin, cinnamic acid, caffeic acid, quinic acid, catechin, coumarin, anthraquinone, and dihydrochalcone). The intrinsic mechanism of phenolic production protects cells against oxidative stress. In addition, members of the family Pteridaceae possess diterpenoids (pterokaurane) and sesquiterpenoids (2,5,7-trimethyl-indan-1-one, pterosin Z, and ptaquiloside), while those of the family Polypodiaceae contain triterpenoids (β-sitosterol, scaphopetalone, neohop-13(18)-ene, and diploptene) [25,26]. Terpenoids are known to be explicitly associated with anticancer and neuroprotective effects [27,28]. Owing to the presence of these bioactive compounds, ferns exhibit a wide range of medicinal properties that are effective in the treatment of various ailments. Moreover, they hold considerable significance in traditional medicine due to their long-standing ethnopharmacological uses. These include the treatment of minor conditions, such as pyrexia, insect bites, burns, blisters, and constipation, as well as more severe health issues, such as urinary tract infections, elevated blood glucose levels, hepatic disorders, parasitic infections, dermatitis, and cancer [25,29]. Despite being a rich source of secondary metabolites, the use of ferns for obtaining these compounds is limited.

The majority of secondary metabolites derived from ferns exhibit similarities to those found in higher plants. However, certain metabolites are unique to pteridophytes. Spectroscopic analysis has been used to elucidate structure, facilitating the identification and quantification of these metabolites from various polar and non-polar organic solvent extracts [30,31]. These unique secondary metabolites, present in different fern families, have been reported to possess medicinal properties, as shown in Table 1. These metabolites, which are exclusive to ferns, differ in their chemical structure and activities. They belong to various classes of secondary metabolites, including sesquiterpenes, triterpenes, flavonoids, phloroglucinol, and lactone, and exhibit antibacterial, antidiabetic, analgesic, anticancer, hepatoprotective, and numerous other properties (Table 1). It is worth noting that certain species with unique secondary metabolites have been traditionally used for treating various ailments. Species within the families Aspleniaceae, Blechnaceae, Dennstaedtiaceae, Dryopteridaceae, Gleicheniaceae, Marattiaceae, Metaxyaceae, Onocleaceae, Osmundaceae, Pteridaceae, and Thelypteridaceae are recognized for their use in folk medicine (Table 2).

The compiled data demonstrate the substantial chemical diversity of ferns (Table 1). Terpenoids represent one of the most prominent groups, with both sesquiterpenes (notably pterosins) and triterpenes identified. These compounds exhibit a wide spectrum of activities, including antibacterial, antifungal, and neuroprotective activities. The frequent occurrence of cytotoxic effect among pterosin derivatives suggests a strong association between their indanone-based structure and their ability to induce apoptosis in cancer cells, often mediated through caspase activation and mitochondrial pathways [32]. By contrast, triterpenes such as fernene and hopane derivatives appear to contribute more prominently to antimicrobial effects, indicating functional divergence within the terpenoid class.

Phloroglucinol derivatives stand out for their specificity toward metabolic and inflammatory targets. Their ability to inhibit enzymes such as protein tyrosine phosphatase 1B (PTP1B) highlights their therapeutic relevance in the management of insulin resistance and type 2 diabetes. Additionally, their suppression of inflammasome-related pathways suggests a role in modulating chronic inflammatory responses.

Flavonoids and flavonoid glycosides demonstrate pronounced multifunctionality, particularly in antiviral and hypoglycemic contexts. The inhibition of viral enzymes, such as neuraminidase in H1N1 influenza, indicates their capacity to interfere with viral replication, while concurrent α-glucosidase inhibition supports their role in glycemic control.

**Table 1:** Secondary metabolites with medicinal properties exclusive to ferns.

No.	Compound	Species	Activity	Mode of Action	References
Terpenes and terpene glycosides					
1	Filicene	*Adiantum cuneatum*	Analgesic	No data available	[33]
2	Filicenal	*Adiantum cuneatum*	Analgesic	No data available	[33]
3	fern-9(11)-ene	*Adiantum lunulatum*	Antibacterial	No data available	[30]
4	fern-9(11)-en-25-oic acid	*Adiantum lunulatum*	Antibacterial	No data available	[30]
5	Adiantone	*Adiantum lunulatum*	Antibacterial	No data available	[30]
6	22,29ξ-epoxy-30-norhopane-13β-ol	*Adiantum lunulatum*	Antibacterial	No data available	[30]
7	Dryofraterpene A	*Dryopteris fragrans* (L.) Schott	Anticancer	No data available	[34]
8	Ancepsone A	*Aleuritopteris anceps*	Anticancer activity	No data available	[35]
9	Creticolacton A	*Pteris cretica*	Exhibit cytotoxicity against colon cancer cell line	No data available	[36]
10	13-hydroxy-2(R),3(R)-pterosin L	*Pteris cretica*		No data available	[36]
11	Creticoside A	*Pteris cretica*		No data available	[36]
12	Spelosin 3-O-β-d-glucopyranoside	*Pteris cretica*		No data available	[36]
13	Bimutipterosin A	*Pteris mutifida* Poir	Cytotoxicity against human leucocythemia carcinoma HL-60 cells	No data available	[37]
14	Bimutipterosin B	*Pteris mutifida* Poir	Cytotoxicity against human leucocythemia carcinoma HL-60 cells	No data available	[37]
15	Obtupterosin A-C	*Pteris obtusiloba*	Cytotoxic activity against cancer cell line	No data available	[38]
16	Decrescensin A	*Pteris decrescens*	Cytotoxic activity against cancer cell line	No data available	[39]
17	Aspleniumside A-C	*Asplenium ruprechtii* Sa. Kurata	Cytotoxic activity against cancer cell line	No data available	[40]
18	(2S,3S)-pterosin C 3-O-b-D-(4′-(E)-caffeoyl)-glucopyranoside	*Pteris multifida*	Cytotoxic activity against cancer cell	Upregulates pro-apoptotic proteins-caspase-9 and procaspase-9	[41]
19	Geopyxin B&E	*Pteris dispar*	Anti-tumor activity		[42]
20	Decrescensin D	*Pteris decrescens*	Anticoagulative activity	No data available	[39]
21	13-chloro-spelosin 3-O-β-d-glucopyranoside	*Ceratopteris thalictroides*, *Hypolepis punctata*, *Nephrolepis multiflora*, and *Pteridium revolutum*	Antidiabetic	No data available	[43]
22	(3R)-Pterosin D 3-O-β-d-(3′-p-coumaroyl)-glucopyranoside	*Ceratopteris thalictroides*, *Hypolepis punctata*, *Nephrolepis multiflora*, and *Pteridium revolutum*	Antidiabetic	No data available	[43]
23	(2R,3R)-Pterosin L 3-O-β-d-(3′-p-coumaroyl)-glucopyranoside	*Ceratopteris thalictroides*, *Hypolepis punctata*, *Nephrolepis multiflora*, and *Pteridium revolutum*	Antidiabetic	No data available	[43]
24	Pteroside Z	*Dennstaedtia scandens*, *Histiopteris incisa*, *Microlepia speluncae*, *Pteridium aquilinum var*. *latiusculum*, *Pteridium revolutum*, *Hypolepis punctata*, *Cer atopteris thalictroides*, *Pteris fauriei*, *Pteris dimidiata*, and *Pteris ensiformis*.	Antidiabetic activity	No data available	[44]
25	Ceratopteroside B				
26	Pterosin D3-O-B-D-glucopyranoside				
27	Ceratopteroside C				
28	2-Hydroxypterosin C				
29	Pterosin A				
30	(2R,3S)-Pterosin C				
31	(2S,3S)-Pterosin C				
32	Pterosin D				
33	Pterosin G				
34	Pterosin I				
35	Pterosin L				
36	Pterosin N				
37	Pterosin X				
38	pteron-14-ene-7α,19α,28-triol	*Adiantum capillus-veneris*	Antifungal activity	No data available	[45]
39	3β,4α,25-trihydroxyfilican	*Adiantum capillus-veneris*	Antifungal activity	No data available	[45]
40	fern-8-ene	*Blechnum orientale*, *Dicranopteris linearis* (Burm.f) *Underw*., *Marattia fraxinea Sm*., and *Microlepia speluncae* (L.) Moore *	Antitrematodal activity	No data available	[31]
41	fern-9(11)-ene	*Blechnum orientale*, *Dicranopteris linearis* (Burm.f) *Underw*., *Marattia fraxinea Sm*., and *Microlepia speluncae* (L.) Moore	Antitrematodal activity	No data available	[31]
42	Ptercresion A	*Pteris cretica* L.	Hepatoprotective	No data available	[46]
43	Ptercresion B	*Pteris cretica* L.	Hepatoprotective	No data available	[46]
44	Ptercresion C	*Pteris cretica* L.	Hepatoprotective	No data available	[46]
45	Pterosin B	*Pteris laeta* Wall. ex Ettingsh.	Neuroprotective activity	Targets the downstream mitochondrial signals; upregulates the expression of nuclear factor-erythroid factor 2-related factor 2 (NRF2) and heme oxygenase-1 (HO-1)	[47]
46	Pterosinsade A	*Pteris laeta* Wall.	Neuroprotective activity	Reduces apoptosis of amyloid precursor protein (APP)-overexpressing neural stem cells; promotes their proliferation and neuronal differentiation; promotes hippocampal neurogenesis, associated with activating the Wnt signaling pathway	[48]
Phloroglucinol derivatives					
47	Trisflavaspidic acid ABB	*Dryopteris crassirhizoma*	Antidiabetic activity	Competitive inhibitor against PTP1B enzyme	[49]
48	Trisflavaspidic acid BBB	*Dryopteris crassirhizoma*	Antidiabetic activity	Competitive inhibitor against PTP1B enzyme	[49]
49	Nortrisflavaspidic acid ABB	*Dryopteris crassirhizoma*	Antidiabetic activity	Competitive inhibitor against PTP1B enzyme	[49]
50	(±)-Dryoptol G	*Dryopteris crassirhizoma* Nakai	Anti-inflammatory	Blocks the formation of inflammasome (suppresses the expression of IL-1β and IL-18; inhibits the expression of NLRP3 and cleaved caspase-1)	[50]
51	(±)-3″-Epi-dryoptol G	*Dryopteris crassirhizoma* Nakai	Anti-inflammatory	Blocks the formation of inflammasome (suppresses the expression of IL-1β and IL-18; inhibits the expression of NLRP3 and cleaved caspase-1)	[50]
52	Yungensins A–E	*Elaphoglossum yungense*	Antibacterial activity	No data available	[51]
53	Lindbergins A–D	*Elaphoglossum lindbergii* (Mett. ex Kuhn) Rosenst.	Antibacterial activity	No data available	[52]
54	Phloropyron A	*Dryopteris championii*	Antibacterial activity	No data available	[53]
55	Pseudoaspidinol A	*Dryopteris championii*	Antibacterial activity	No data available	[53]
56	Dryocrassoid A–J	*Dryopteris crassirhizoma* Nakai	Antiviral activity	No data available	[54]
57	Filixic acid ABA	*Dryopteris crassirhizoma* Nakai	Antiviral activity	No data available	[50]
58	Paleacenins A&B	*Elaphoglossum paleaceum* (Hook. & Grev.) Sledge	Cytotoxic activity against cancer cell line	No data available	[55]
59	Wallichins A–D	*Dryopteris wallichiana* (Spreng.) Hyl.	Nematocidal activity	No data available	[56]
δ-Lactone Glycosides					
60	Angiopteroside	*Angiopteris helferiana* C.Presl, *Angiopteris evecta* Hoffm.	Anti-adipogenic and cytotoxicity against lung cancer cell	No data available	[57,58]
61	Osmundalin	*Todea Barbara* (L.) T. Moore, *Osmunda japonica* Thunb., *Angiopteris caudatiformis*	Antifeedant	Activation of R receptor cells	[59,60]
Flavonoids and flavonoid glycosides					
62	Matteuorienates A–C	*Matteuccia orientalis* Trev.	Aldose reductase inhibition	No data available	[61]
63	Quercetin 7,3′,4′-trimethoxy	*Blechnum orientale*, *Dicranopteris linearis* (Burm.f) *Underw*., *Marattia fraxinea Sm*., and *Microlepia speluncae* (L.) Moore	Antitrematodal activity	No data available	[31]
64	3′-Hydroxy-5′-methoxy 6,8-dimethyl huazhongilexone	*Pentarhizidium orientale* (Hook.) Hayata	Antiviral activity	Neuraminidase (NA) inhibition of H1N1 influenza virus	[62]
65	Matteflavoside G	*Matteuccia struthiopteris* (L.) Todar	Antiviral activity	Neuraminidase (NA) inhibition of H1N1 influenza virus	[63]
66	Matteucin	*Pentarhizidium orientale* (Hook.) Hayata; *Matteuccia intermedia* C.Chr	Antiviral activity and hypoglycemic effect	Neuraminidase (NA) inhibition of H1N1 influenza virus and α-Glucosidase inhibitory activity	[62,64]
67	Methoxymatteucin	*Pentarhizidium orientale* (Hook.) Hayata; *Matteuccia intermedia* C.Chr	Antiviral activity and hypoglycemic activity	Neuraminidase (NA) inhibition of H1N1 influenza virus and α-Glucosidase inhibitory activity	[62,64]
68	Cyrtominetin	*Matteuccia intermedia* C.Chr	Hypoglycemic activity	α-Glucosidase inhibitory activity	[64]
69	3′-hydroxymatteucinol	*Matteuccia intermedia* C.Chr	Hypoglycemic activity	α-Glucosidase inhibitory activity	[64]
70	2′-hydroxymatteucinol	*Matteuccia orientalis* Trev.	Hypoglycemic activity	No data available	[65]
71	2-deprenyl-5-*O*-methyl-7-hydroxy-rheediaxanthone B	*Metaxya rostrata* (Kunth) C. Presl	Cytotoxic activity against cancer cell line	No data available	[66]
72	2-deprenyl-5-O-methyl-7- methoxy-rheediaxanthone B	*Metaxya rostrata* (Kunth) C. Presl	Cytotoxic activity against cancer cell line	No data available	[66]
73	2-deprenyl-6-O-methyl-7-hydroxy-rheediaxanthone B	*Metaxya rostrata* (Kunth) C. Presl	Cytotoxic activity against cancer cell line	No data available	[66]
74	Abacopterins A and C	*Pronephrium penangianum* (Hook.) Holtt	Cytotoxic activity against tumor cell line	No data available	[67]
75	Eruberin B	*Pronephrium penangianum* (Hook.) Holtt	Cytotoxic activity against tumor cell line	No data available	[67]
76	Triphyllin A	*Pronephrium penangianum* (Hook.) Holtt	Cytotoxic activity against tumor cell line	No data available	[67]
Other Phenolic					
77	Dryofracoumarin A	*Dryopteris fragrans* (L.) Schott	Anticancer activity	No data available	[68]
78	Albicanol	*Dryopteris fragrans* (L.) Schott	Anti-tumor activity	No data available	[69]
79	Liglaurates A–D	*Drynaria roosii* Nakaike	Cytotoxic activity against tumor cell line	No data available	[70]
80	Drycrasspherols A	*Dryopteris crassirhizoma* Nakai	Antiviral activity	No data available	[71]
81	Cibotiumbaroside D	*Cibotium barometz* (L.) J. Sm.	Hepatoprotective activity	No data available	[72]

* The authors of the Latin names are cited as they were written in the article.

Phenolic compounds further contribute to the pharmacological profile of these metabolites, particularly through cytotoxic, hepatoprotective, and antiviral activities. Their antioxidant properties and ability to interact with cellular signaling pathways enhance their relevance in disease prevention and therapy. Notably, compounds exhibiting hepatoprotective effects suggest potential applications against liver disorders, while antiviral phenolics reinforce the importance of ferns as a source of bioactive molecules with broad-spectrum activity.

**Figure 1:**
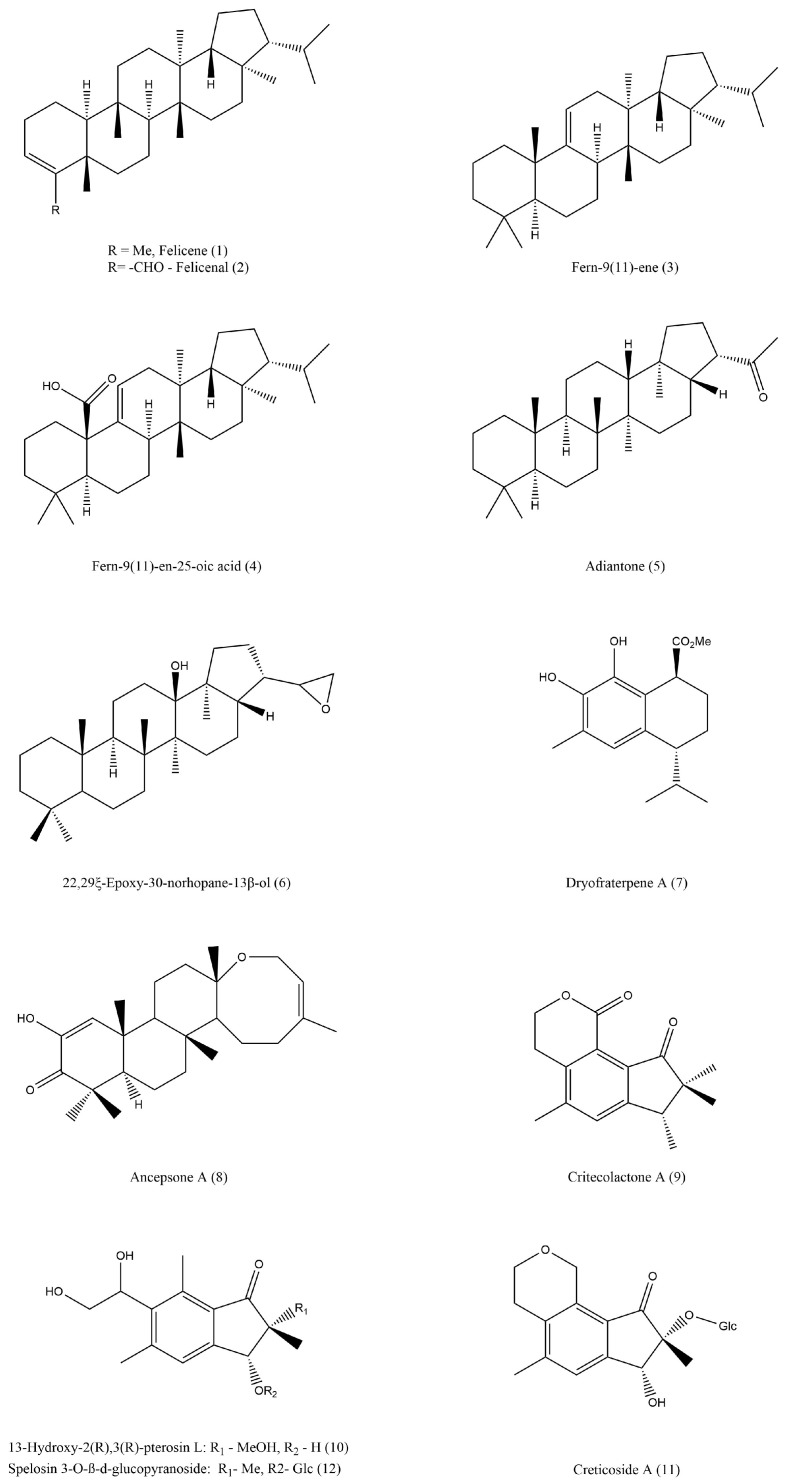
Chemical structures of compounds **1**–**12** as discussed in Table 1.

**Figure 2:**
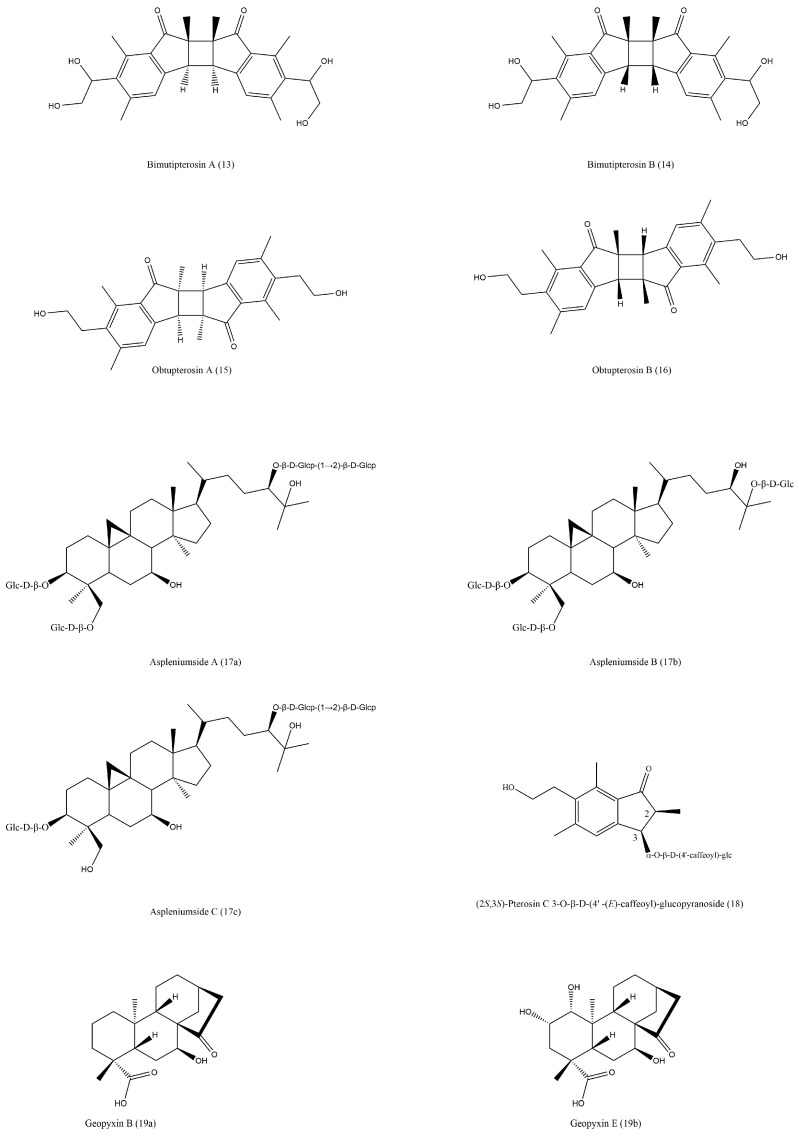
Chemical structures of compounds **13**–**19** as discussed in Table 1.

**Figure 3:**
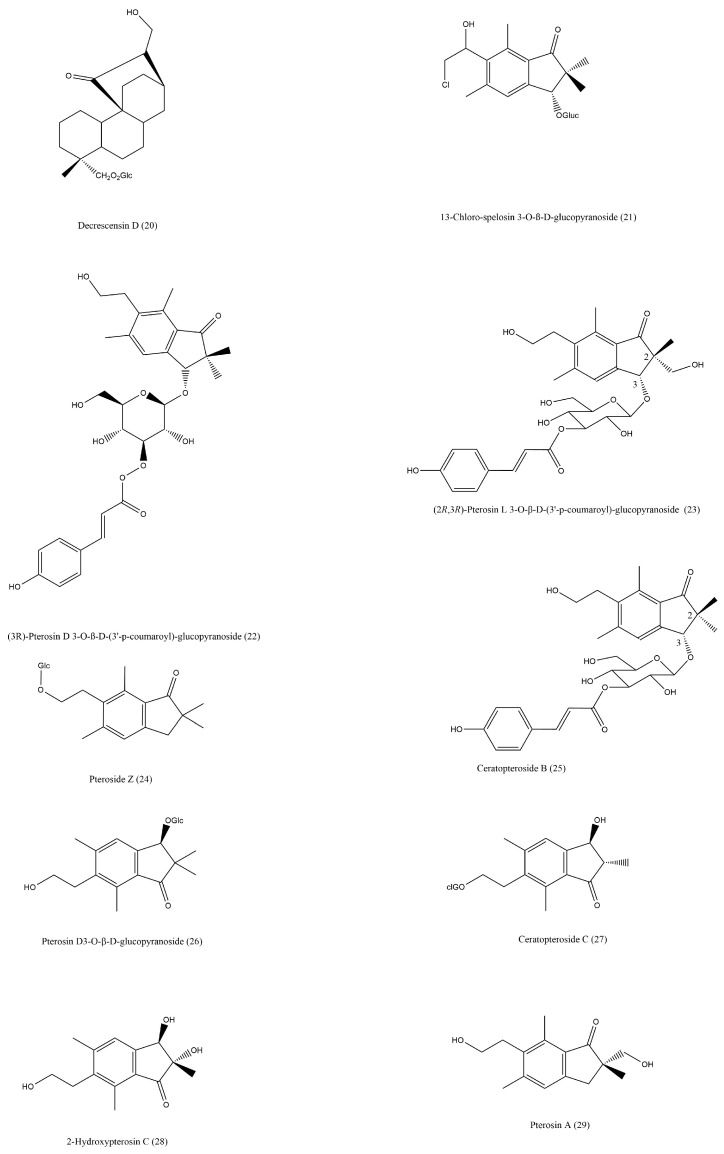
Chemical structures of compounds **20**–**29** as discussed in Table 1.

**Figure 4:**
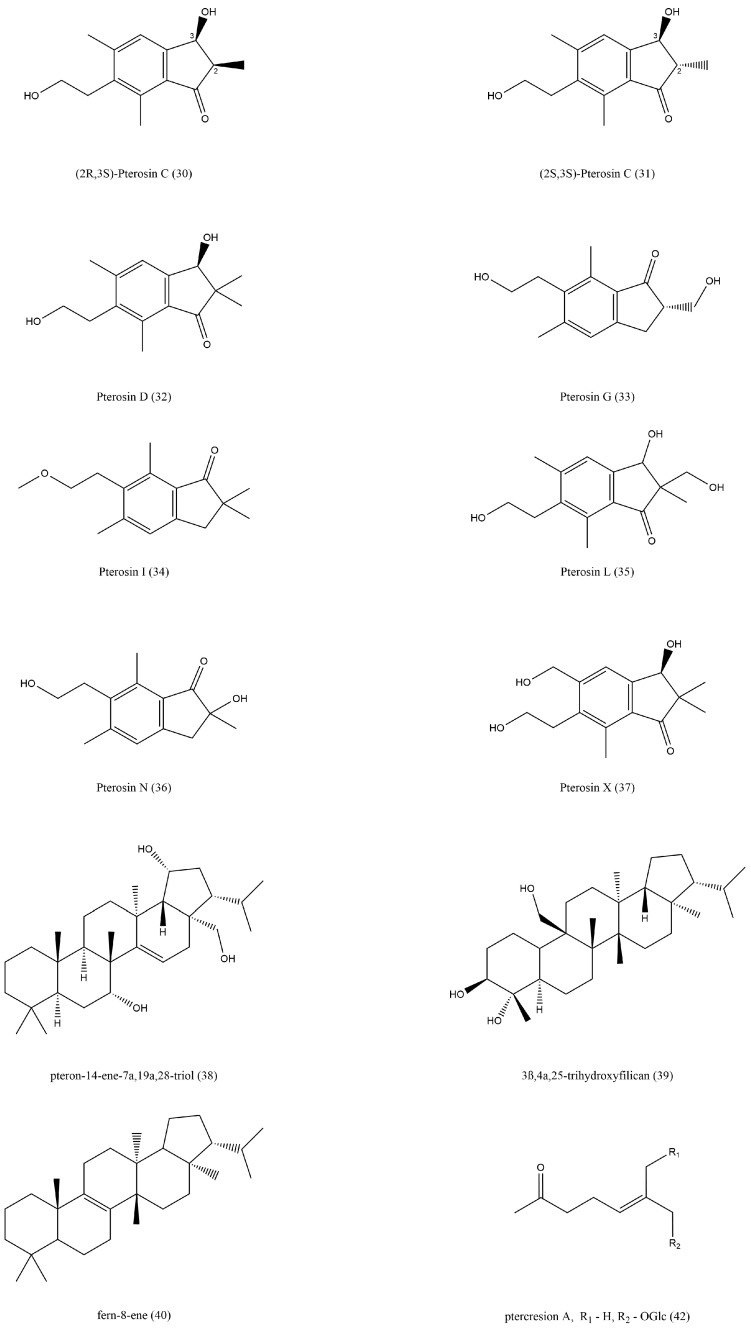
Chemical structures of compounds from **30**–**42** as discussed in Table 1.

**Figure 5:**
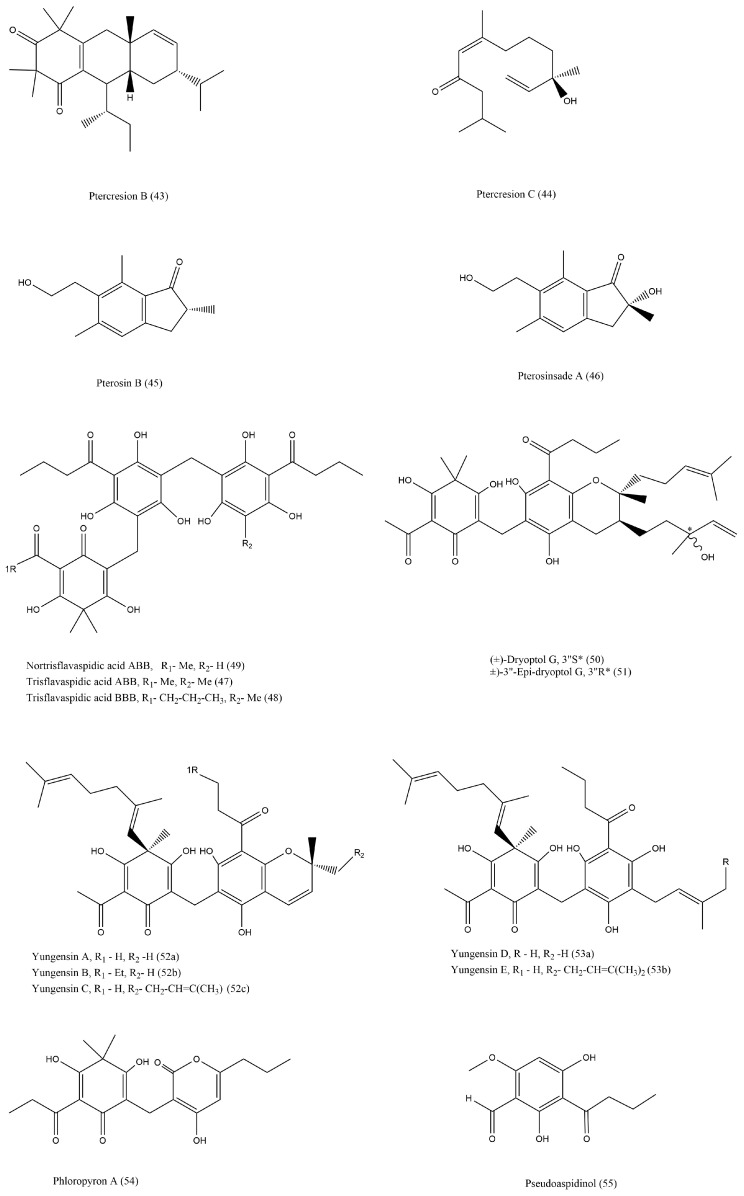
Chemical structures of compounds **43**–**55** as discussed in Table 1.

**Figure 6:**
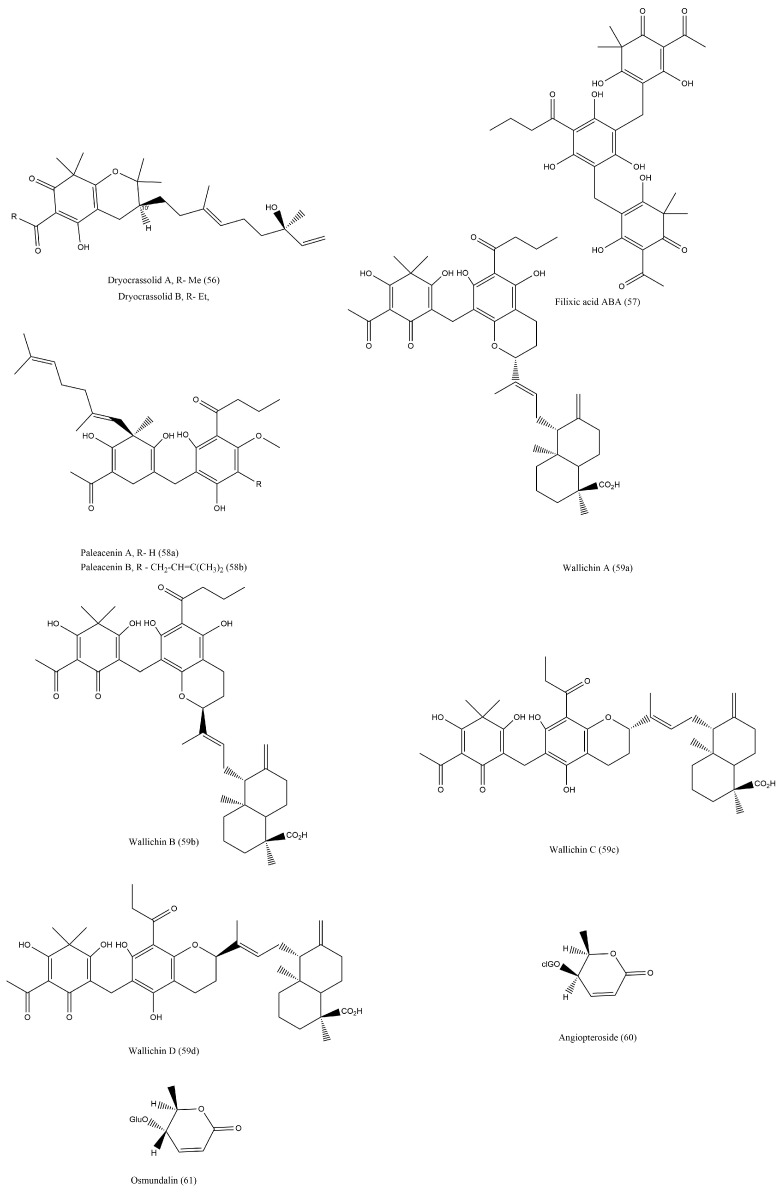
Chemical structures of compounds **56**–**61** as discussed in Table 1.

**Figure 7:**
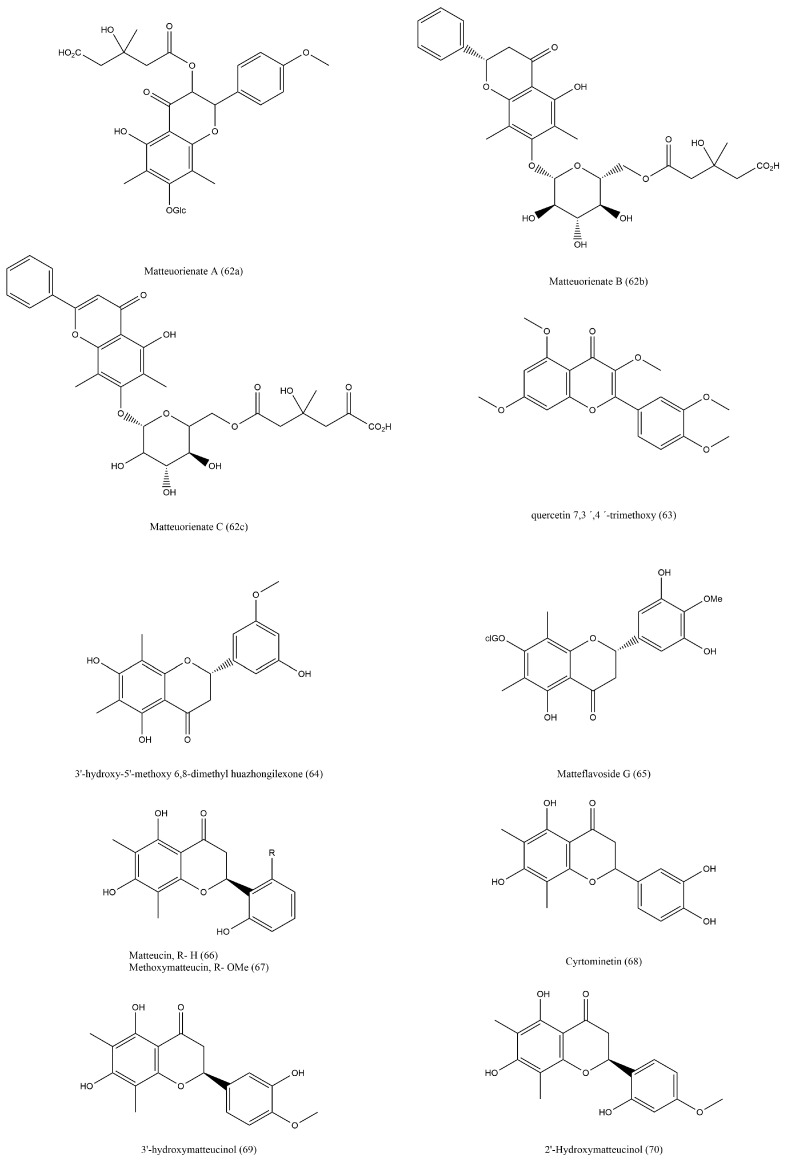
Chemical structures of compounds **62**–**70** as discussed in Table 1.

**Figure 8:**
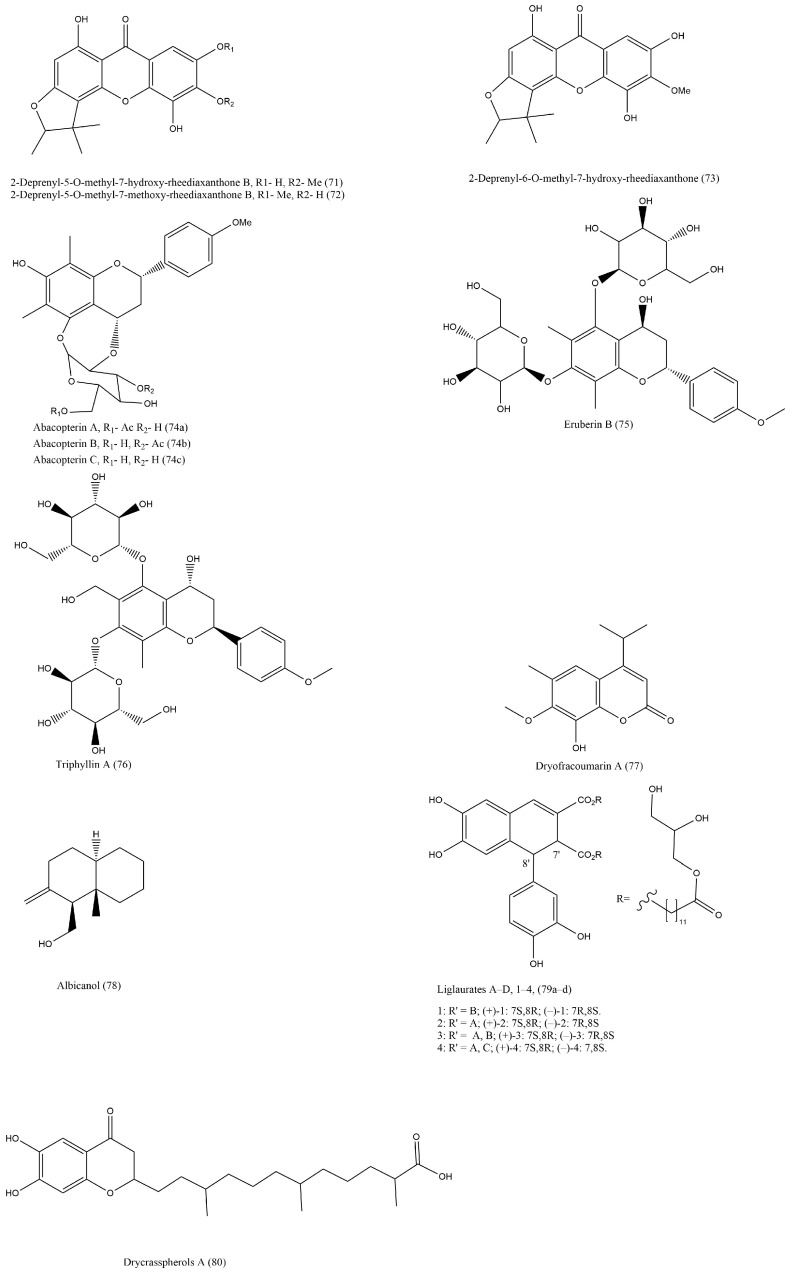
Chemical structures of compounds from **71**–**80** as discussed in Table 1.

Overall, a key pattern emerging from the dataset is the prevalence of enzyme inhibition and signaling pathway modulation as dominant mechanisms of action. Pathways such as the Nrf2 signaling pathway and apoptosis-related cascades are recurrently targeted, indicating that these metabolites act through well-defined molecular mechanisms rather than nonspecific toxicity. This enhances their suitability as lead compounds for drug development. Despite these promising observations, the lack of standardized potency data and incomplete mechanistic characterization for several compounds limits direct comparison and clinical translation. The chemical structures of the compounds listed in Table 1 are shown in Figure 1, Figure 2, Figure 3, Figure 4, Figure 5, Figure 6, Figure 7 and Figure 8, numbered and arranged according to their order of appearance in the table.

**Table 2:** Ferns possessing unique secondary metabolites that are recognized for their traditional medicinal uses.

Sr. No.	Family	Species	Traditional Medicinal Use		References
1	Aspleniaceae	*Asplenium ruprechtii*	Hemiplegia, promote blood circulation, uterine bleeding, traumatic bleeding, anti-inflammatory, and hemostasis	Northern Provinces of China	[73,74]
2	Blechnaceae	*Blechnum orientale*	Boils, reproductive control agent, antihelmintic, typhoid, intestinal worms, and bladder complaints	Kadazan/Dusun communities in Sabah, Malaysia; Papua New Guinea	[75,76,77]
3	Dennstaedtiaceae	*Hypolepis punctata*	Infection, digestive disorders	Traditional Chinese Medicine	[78]
		*Pteridium revolutum*	Antipyretic and insect repellent	Traditional Chinese Medicine	[79]
		*Pteridium aquilinum*	Antibacterial, diuretic	Kosovar, Albanian Alps	[80]
4	Dryopteridaceae	*Dryopteris championii*	Cold, asthma, hemafecia, dysmenorrhea	Traditional Chinese Medicine	[53]
		*Dryopteris crassirhizoma*	Tapeworm infestation and mumps	Traditional Korean Herbal Medicines	[81]
		*Dryopteris fragrans*	Psoriasis, arthritis, rash, dermatitis, barbiers, antimicrobial, and anticancer	Traditional Chinese Medicine	[82]
5	Gleicheniaceae	*Dicranopteris linearis*	Fever, asthma, external wound, and ulcers	Traditional Chinese Medicine	[83]
6	Marattiaceae	*Angiopteris helferiana*	Scabies	China, India (Ayurveda), Nepal	[84]
		*Angiopteris evecta*	Snake bite, diuretic, antipyretic, analgesic, and antidiarrheal	‘Tangsa’ an ethnic Sino-Burmese community, Arunachal Pradesh, India	[58,85]
7	Metaxyaceae	*Metaxya rostrata*	Gastrointestinal disorders	Corcovado Parque National in Costa Rica	[86,87]
8	Onocleaceae	*Matteuccia orientalis*	Hemostasis and relieving ostalgia	Traditional Chinese Medicine	[88]
		*Pentarhizidium orientale* (Synonymous with *Matteuccia orientalis*)	Diuretic and helminthic	Korean Folk Medicine	[62]
		*Matteuccia struthiopteris*	Dysentery and prevention of influenza	Traditional Chinese Medicine	[63]
9	Osmundaceae	*Osmunda japonica*	Antiviral, antiherpetic, hemostasis, and pesticidal	Traditional Chinese Medicine	[89]
10	Pteridaceae	*Adiantum capillus-veneris*	Astringent, expectorant, emmenagogue, catarrhal and menstrual problems, cough, cold, and bronchial diseases	Ayurveda (traditional medicine system of India), Italy, Peru, Palestine, Pakistan, Portugal, Spain, Greece, and China	[77,90]
		*Adiantum cuneatum*	Diuretic, expectorant, emollient, used for coughs, urinary disorders, alopecia, and menstrual difficulties	Argentina, Peru (Quechua speaking communities in Ancash region), Brazil (Atlantic Forest region)	[33,90]
		*Adiantum lunulatum*	Snake bite, bronchitis, and asthma	Ethnomedicine of Garhwal Region, Uttarakhand, India	[82]
		*Pteris cretica*	Jaundice, hepatitis, enteritis, bacillary dysentery, turbid conditions, vomiting, bleeding, hematochezia, hematuria, tonsillitis, mumps, carbuncle, and eczema	Traditional Chinese Medicine	[91,92]
		*Pteris decrescens*	Enteritis, jaundice, hepatitis, bloated sores, and epistaxis	Miao Traditional Medicine	[39]
		*Pteris laeta*	Inflammation, dysentery, relaxing tendons and activating collaterals, promoting the union of fracture healing, and relieving spasms, rheumatism, chronic hepatitis, dysentery, and nervous system diseases	Miao Medicine	[47]
		*Pteris mutifida*	Tonsillitis, parotitis hepatitis, eczema, hematemesis, enteritis, and diarrhea	Traditional Chinese Medicine	[92,93]
11	Thelypteridaceae	*Pronephrium penangianum*	Rheumatoid arthritis, strain injury, traumatic injury upper respiratory tract infections, dysentery, and edema	Tujia Ethnomedicine	[67]

## 4. Influence of Abiotic Stress Factors on Secondary Metabolite Production in Ferns

### 4.1. Light

Recent studies investigating the role of light as a stimulator have indicated that light intensity and quality are fundamental to the induction of secondary metabolite production. In the quest to enhance secondary metabolite production within plants, relying solely on photosynthetically active radiation (PAR) within the wavelength range of 400–700 nm does not help in achieving optimal outcomes. It is imperative to explore physiologically active radiation at λ 300–800 nm to truly unlock the metabolic potential of plants. Within this range, radiation stimulates different photoreceptors. In higher plants, the response to various light spectra is governed by photoreceptors, namely the ZTL/FKF1/LKP2 complex, UV RESISTANCE LOCUS8 (UVR8), phototropins, phytochromes, and cryptochromes [94,95,96]. Therefore, upon reception of different spectra/intensities of light, the aforementioned receptors activate a signaling cascade, which alters plant biochemistry by producing different compositions of secondary metabolites, such as cyanogenic glycosides, alkaloids, terpenes, tannins, and phenols, to combat stress (Table 3).

#### 4.1.1. Light Spectrum

LED technology incorporating advanced semiconductor materials is being progressively adopted across diverse sectors, particularly in agriculture to promote plant growth [111]. As a substitute for conventional lighting, LEDs provide accurate modulation of light conditions, facilitating plant development by emitting specific spectral regions that correspond to plant photoreceptors. Furthermore, LEDs are available in a spectrum of colors, such as violet, blue, green, yellow, red, far-red, and white. Red (600–700 nm) and blue (400–500 nm) light demonstrate the highest photosynthetic photon efficacy values, while green light (500–600 nm) displays significant penetrance [96]. This characteristic enables the efficient regulation of photosynthetically active and photomorphogenic radiation, thereby optimizing plant growth and metabolic processes. In the tree fern *Cyathea delgadii*, a spectral combination of 35% red, 15% blue, and 50% UV (RBUV-400 nm), as well as 100% blue light (B-430 nm), had remarkable effects on the production of sporophytes in internode explants [112]. Furthermore, various LED light conditions induced distinct morphogenetic responses during both gametophytic and sporophytic developmental stages in ferns. Specifically, 70% red and 30% blue were associated with root development; 100% red light (670 nm) influenced spore germination and leaf elongation; and white light (1:1:1 2700 K:4500 K:5700 K) and fluorescent light (400 foot-candles) positively influenced gametophytic growth in *Cyathea delgadii* and *Onoclea sensibilis*, respectively [112,113,114].

Cryptochromes (CRY1, CRY2, CRY3) and phototropins (PHOT1, PHOT2) in *Arabidopsis* are sensitive to blue/UV-A light [94]. CRY1, CRY2, PHOT1, and PHOT2 detect blue light and initiate signaling. Activated CRY1 and CRY2 translocate to the nucleus to undergo phosphorylation, hindering COP1–SPA1 interaction and activating HY5, which modulates the gene expression of secondary metabolite biosynthesis pathways [95]. Recently, blue-light photoreceptor genes, namely cryptochrome genes 4 and 5 (CRY4 and CRY5), were isolated from the fern *Adiantum capillus-veneris* [113,115]. Phytochromes exist in the inactive form (Pr) and the active form (Pfr) and are located in the cytoplasm, with the latter present in the nucleus in an interconvertible state [94]. Upon stimulation by red light, the Pr form in the cytoplasm is activated to the Pfr form and is subsequently transported to the nucleus, where it initiates its protein kinase activity, resulting in autophosphorylation. Once activated, it regulates the gene expression of secondary metabolite biosynthesis pathways [95,96]. Consequently, red and blue light play pivotal roles in the biosynthesis of secondary metabolites both in vivo and in vitro. These wavelengths of light enhance the biosynthesis of alkaloids, terpenoids, and flavonoids, whether applied individually or in combination (Table 3).

#### 4.1.2. UV Radiation

From among three types of ultraviolet radiation, UV-A (315–400 nm), UV-B (280–320 nm), and UV-C (100–280 nm), only UV-A penetrates the stratosphere and reaches Earth’s canopy, while UV-B and UV-C are absorbed by the stratosphere at different ranges [116]. In the context of UV receptivity, ferns exhibit changes in the developmental axis, reversal of polarity in protonema, and swelling of rhizoid and protonema cells [117]. Along the same line, studies on polarotropic response in *Dryopteris filix-mas* (L.) Schott demonstrated the presence of phytochrome action in the blue and UV regions [118]. Recent studies have reported that cryptochromes, phytochromes, and the ZTL/FKF1/LKP2 receptor complex are responsible for the perception UV-A light, whereas the UVR8 photoreceptor is sensitive to UV-B light [95].

Thus, photoreceptors identify specific wavelengths of light and propagate the signal through a cascade that modulates gene expression, culminating in distinct physiological responses. This is also the reason for the activation of secondary metabolite pathways. The process encompasses the transition of UVR8 from its inactive to active state, ensued by the sequestration of CONSTITUTIVELY PHOTOMORPHOGENIC-1 (COP1), an E3 ubiquitin ligase, by UVR8, which consequently stabilizes ELONGATED HYPOCOYL 5 (*HY5*), a transcription factor that activates genes associated with morphogenesis and metabolic pathways [95]. According to Yeshi et al. [8], plants synthesize UV-absorbing flavonoids, which are pivotal in quenching reactive oxygen species (ROS) or mitigating UV penetration, thus reducing photooxidative damage. In a study on *Acrostichum danaeifolium*, highly developed grana organization on the thylakoid membrane and increased abundance of starch grains in chloroplasts was observed after UV-B exposure [103]. The high accumulation of starch grains in chloroplasts corresponded with an increased carbohydrate reserve, thereby increasing the concentrations of carbon-based secondary metabolites, such as phenolics [103]. A similar result was observed for *Azolla microphylla*, wherein an increase in phenolic content was observed (Table 3).

### 4.2. Temperature

Maintaining membrane fluidity within plant cells is essential for various biological functions. Heat and cold stresses affect the fluidity of cellular membranes, which is detected by plasma membrane proteins. Cold stress leads to calcium influx and the activation of calcium-responsive kinases, resulting in cold-responsive gene expression [119]. Heat stress activates heat shock proteins for protein homeostasis. Plant heat stress transcription factors (HSFs) are activated by the dissociation of HSP70 and HSP90 chaperones, initiating heat stress responses [120]. ROS signaling is also associated with heat stress. In angiosperms, heat stress is not only associated with the production of ROS and increased synthesis of isoprene units [121,122] but also increased levels of isoprenoid precursors [122,123,124].

Therefore, terpenoids are predominant secondary metabolites produced by plants in response to heat stress. This phenomenon can also be attributed to the fact that high temperature denatures flavonoids [125,126,127]. Specifically, there is a divergence of carbon flow toward the non-mevalonate pathway instead of its utilization in photosynthesis [8]. This was evident in a study on the leatherleaf fern *Rumohra adiantiformis* (Forst.) Ching, wherein high temperature (30 °C day/25 °C night) improved growth and induced earlier sori production and lower light-saturated net CO_2_ assimilation rates compared to low temperature (20 °C day/15 °C night) [128]. With regard to isoprene production, in a study on *Dicksonia antarctica* Labill, *Thelypteris decursive-pinnata* (Van Hall) Ching, and *Pelazoneuron kunthii* (Desv.) A.R.Sm. & S.E.Fawc., high temperature (35–36 °C) caused maximum emissions. High temperature elevates the vapor pressure of isoprene, thereby enhancing the concentration gradient that facilitates diffusion from the plant [101]. Moreover, in *D. antarctica*, the critical temperature (47 °C) did not affect the accumulation of protective compounds like zeaxanthin, α-tocopherol, violaxanthin, antheraxanthin, neoxanthin, and lutein but positively influenced the accumulation of α- and β-carotenes due to their role in establishing membrane stability [100].

### 4.3. Drought

Drought is one of the most serious and frequent abiotic stressors, and in the face of global warming, drought incidents are expected to escalate. Drought stress can cause severe damage in plants. Plants have various mechanisms for surviving water scarcity. Accumulation of reactive oxygen species (ROS) is a physiological response to drought stress. Increased levels of ROS, such as hydrogen peroxide (H_2_O_2_), induce the peroxidation of membrane lipids, oxidation of proteins, and DNA damage [24]. An increase in H_2_O_2_ concentration was registered in *Matteuccia struthiopteris* (L.) Todar and *Athyrium multidentatum* during drought stress [107]. To mitigate such conditions, an upregulation in the production of flavonoids has been demonstrated due to their antioxidant properties [24]. The underlying metabolic background behind the stress-driven increase in secondary metabolite production in medicinal plants was described in ref. [129]. The initial reduction in water availability causes stomata to close, leading to a significant drop in CO_2_ uptake. Consequently, the use of reduction equivalents (NADPH + H^+^) for carbon fixation through the Calvin cycle is greatly reduced, resulting in excess NADPH + H^+^. This drives metabolic pathways to produce highly reduced substances, such as isoprenoids, phenols, and alkaloids.

In *Athyrium multidentatum* and *Matteuccia struthiopteris* (L.) Todar, an initial period of drought stress increased antioxidant enzyme activity and the production of secondary metabolites like total phenols, flavonoids, and proanthocyanidins, while it reduced H_2_O_2_ levels [107]. Similarly, in *Polypodium vulgare* rhizomes cultured in vitro, increased levels of phenolic compounds were observed after drought stress imposed by immersion in mannitol [108]. In addition, there was an increase in the accumulation of sugars, such as mannitol, trehalose, and glucose, as well as amino acids. Similarly, in *Athyrium nipponicum* cv. Metallicum, drought stress enhanced the accumulation of reducing sugars in leaves [106].

Moreover, metabolomic studies of drought stress in *Adiantum nelumboides* leaves reported stress-induced upregulation of primary and secondary metabolites, including amino acids and derivatives, nucleotides and derivatives, phenolic acids, alkaloids, and flavonoids [109].

### 4.4. Nutrients

Both macro- and micro-nutrients are essential for plant growth and development. In tissue cultures, sucrose serves as an essential carbon source in culture media, supplementing carbon reserves and enhancing photosynthetic capacity, which is otherwise impacted by reduced gaseous exchange and irradiance. Each developmental stage necessitates a specific concentration of sucrose. Minor alterations can influence progression to subsequent stages. While 1% or 3% sucrose exerts a minimal impact on tissue development in angiosperms, these concentrations may pose stress to developing fern gametophytes. In parallel, the formation of reactive oxygen species (ROS) in *Diplazium maximum* was reported at 3% sucrose, whereas cell death and necrosis were observed at 6% sucrose. Moreover, the presence of elevated ROS levels consequently stimulated the synthesis of secondary metabolites in gametophytes of *Diplazium maximum* [110]. In *Adiantum reniforme* var. *sinense*, initial gametophyte development and spore germination required 15 g dm^−3^ sucrose, whereas later stages of gametophyte development required 30 g dm^−3^. However, sucrose concentrations ranging from 45 to 60 g dm^−3^ exhibited adverse effects by impeding the growth and development of gametophytes [130]. Conversely, in the gametophytes of *Osmunda regalis* and *Pteris ensiformis*, the mere presence of sucrose was found to inhibit development and induce necrosis [131].

Modulation of nitrogen (nitrate/ammonium), potassium, phosphate, and calcium facilitates spore germination and promotes growth. For example, such modulation regulates the initiation of foliar organs at the stem apex, as well as frond and secondary branch development, in *Pteridium aquilinum* [132]. Similarly, the elimination of NH_4_NO_3_ and 25% nitrogen depletion in MS medium optimized the yield of *Adiantum capillus-veneris* sporophytes [133]. Furthermore, *Polystichum polyblepharum*, *Polypodium vulgare*, and *Onoclea sensibilis* grown in the presence of nutrient-rich camelina press cakes (cakes derived from *Camelina sativa* seed oil) demonstrated notable increases in growth, chlorophyll levels, and carotenoid content, with *Polystichum polyblepharum* also exhibiting a significant increase in flavonoid content [134]. A connection can be drawn between these metabolic pathways via phenylalanine ammonia lyase (PAL) activity, wherein the enzymatic activity influences flavonoid production [135,136]. Phosphorus is a constituent of ADP and ATP in primary metabolism. In secondary metabolism, its deficiency leads to anthocyanin production and reduced development [137,138]. The lack of Mg, which is involved in chlorophyll formation, elevates reactive oxygen species concentrations and positively influences carotenoid synthesis [139,140,141]. Cu serves as a cofactor in the functioning of oxygenases and oxidase enzymes linked to secondary metabolism [142,143].

### 4.5. NaCl Concentration

ROS and Ca^2+^ are secondary messengers that are critical for modulating the initial response to stress, leading to the fine-tuning of secondary metabolite biosynthesis [144,145]. The activation of ROS and Ca^2+^ is triggered by ionic and osmotic stresses due to high salt concentrations. Initially, ionic stress sensors sense an increase in ionic concentration. This increases Ca^2+^ levels. Ca^2+^ then binds to SOS3 protein (encoded by the Salt Overly Sensitive 3 (*SOS3*) gene), facilitating its interaction with the SOS2 kinase to form a complex that activates the SOS1 membrane protein, thereby managing ionic stress [146].

Variations in cytoplasmic Ca^2+^ concentrations are perceived by various calcium sensors. including calcium-dependent protein kinases (CDPKs), which engage downstream signaling components, promote the phosphorylation of phenylalanine ammonia lyase (PAL) enzyme, and modulate secondary metabolite biosynthetic pathways [144,146,147]. The effect depends on the salt concentration, duration of exposure, and species under consideration. This activation of PAL was correlated with the accumulation of phenolic compounds in *Phyllitis scolopendrium* and *Ceterach officinarum* after short-term exposure to low NaCl concentrations. *Asplenium viride*, on the contrary, inherently maintained elevated levels of phenolics, allowing it to endure even greater salt levels [105]. Phenolics are potent antioxidants. The underlying mechanism in higher plants is attributed to the respiratory burst oxidase homolog (*RBOH*) gene family, which is regulated and activated by CDPK and encodes the enzyme nicotinamide adenine dinucleotide [145]. This enzyme facilitates the production of reactive oxygen species (ROS) in the extracellular space and consequently augments ROS levels within the cell. Increased ROS levels positively influence secondary metabolite biosynthetic pathways, thereby protecting plants against unfavorable conditions [148,149].

### 4.6. Interactive Effects of Combined Abiotic Stresses on Secondary Metabolite Production, Hormone Regulation, and the Role of ROS and Ca^2+^ Signaling

In nature, plants, including ferns, rarely encounter a single abiotic stressor in isolation. Concurrent stresses, such as high light intensity combined with drought, salinity accompanied by temperature extremes, or nutrient deficiency under altered light spectra, are common, especially under climate change scenarios. These combined stresses can produce interactive effects on secondary metabolite biosynthesis that are not simply additive but often synergistic, antagonistic, or qualitatively distinct from single-stress responses.

An analysis of the studies summarized in Table 3 reveals clear overlapping induction of the production of specific secondary metabolites across different abiotic stressors in ferns. Flavonoids and total phenolics are the most consistently upregulated metabolites, with their contents increasing under light stress (e.g., 35% full sunlight in *Matteuccia struthiopteris*, *Athyrium multidentatum*, and *Osmunda cinnamomea*), salinity (e.g., 100 mM NaCl in *Athyrium nipponicum*, *Dryopteris erythrosora*, and several species within the family Aspleniaceae), and drought (e.g., 5–17% soil moisture in *Athyrium multidentatum*, *Matteuccia struthiopteris*, and *Adiantum nelumboides*). Similarly, proanthocyanidins and carotenoids show overlapping accumulation under drought and salinity, while isoprene emissions increase under both light and high-temperature regimes in ferns of the families Dicksoniaceae and Thelypteridaceae. This convergence strongly suggests the existence of shared regulatory modes that integrate multiple stress signals to prioritize the biosynthesis of antioxidant and protective compounds (particularly flavonoids and phenolic acids), which help mitigate oxidative damage regardless of the primary stressor.

In addition to metabolite-level responses, endogenous phytohormonal regulation plays a significant role in combating abiotic stress, although the underlying mechanisms remain less explored than in angiosperms. Recent evidence [150] from the species *Pteris cretica* demonstrates that ferns possess complex phytohormone profiles, including both growth- and stress-related hormones, which are dynamically regulated under environmental stress conditions such as arsenic exposure. A comprehensive metabolomic analysis identified more than 20 phytohormones and their analogs in fronds and roots, indicating active endogenous hormone biosynthesis and transport. Notably, interactions between jasmonic acid (JA) and abscisic acid (ABA) were observed in the arsenic-hyperaccumulating variety, suggesting coordinated hormonal feedback involved in oxidative stress mitigation. This provides strong support for the existence of de novo phytohormone synthesis and signaling networks in ferns under abiotic stress, particularly involving the JA and ABA pathways. This hormonal regulation is closely linked to secondary metabolite production through metabolic crosstalk. For example, in vitro and ex vitro studies show that jasmonic acid induces the production of volatile organic compounds, including green leaf volatiles and terpenoids, in several fern species, such as *Cyathea dealbata* and *Cyathea medullaris*, reflecting the activation of rapid defense signaling pathways [151]. By contrast, auxin–cytokinin combinations (e.g., 2,4-D + BAP in *Cyathea delgadii*) promote the accumulation of phenolic and flavonoid compounds, including quercetin and kaempferol derivatives, indicating stimulation of the phenylpropanoid pathway [152]. Therefore, stress-induced phytohormones not only function in signaling but also regulate metabolic flux toward specific classes of secondary metabolites, such as terpenoids or phenolics, depending on the hormonal context.

At the molecular level, these integrated responses are coordinated by central signaling hubs. In particular, reactive oxygen species (ROS) and calcium ions (Ca^2+^) serve as central hubs mediating these interactive effects. Virtually all abiotic stressors (light/UV, temperature extremes, drought, salinity, and mineral imbalance) trigger a rapid ROS burst, primarily via plasma-membrane NADPH oxidases (RBOHs) and photosynthetic electron transport imbalances [8]. In ferns, elevated H_2_O_2_ levels have been documented under drought in *Matteuccia struthiopteris* and *Athyrium multidentatum* and are likely to occur under other stresses as well [115]. ROS act both as damaging agents and signaling molecules; at moderate levels they activate mitogen-activated protein kinase (MAPK) cascades and transcription factors (MYB, WRKY, and bHLH), which upregulate phenylalanine ammonia-lyase (PAL) and key genes in the flavonoid and terpenoid pathways [8,153,154,155]. When multiple stresses coincide, ROS signaling pathways can interact, sometimes resulting in enhanced accumulation of overlapping metabolites such as flavonoids, which contribute to ROS scavenging and membrane stabilization.

Closely interacting with ROS signaling, calcium signaling provides another layer of integration. Stress-induced Ca^2+^ influx (via channels activated by ROS, mechanical changes, or osmotic shifts) is decoded by calcium-dependent protein kinases (CDPKs) and calcineurin B-like proteins (CBLs), which in turn phosphorylate downstream targets, including PAL and terpenoid synthases. In higher plants, combined stresses frequently produce unique Ca^2+^ signatures (amplitude, frequency, and spatial distribution) that fine-tune secondary metabolism differently than single stresses [144,145,146,147]. Although direct evidence in ferns is still limited, the presence of conserved Ca^2+^-signaling components and the observed cross-stress induction of phenolics suggest that similar decoding mechanisms operate in pteridophytes.

Importantly, interactive effects are not always beneficial for metabolite yield. Antagonistic outcomes (e.g., excessive ROS leading to cellular damage and reduced biosynthetic enzyme activity) can occur under severe combined stress, underscoring the need for carefully optimized multi-stress regimes. Future multi-omics studies (transcriptomics + metabolomics under combined vs. single stresses) in key fern model species will be crucial for mapping these interactive regulatory networks and identifying “sweet spot” combinations that maximize the accumulation of unique fern-specific metabolites (e.g., pterosins, phloroglucinols, and terpene glycosides) while minimizing growth penalties.

## 5. Future Scope and Conclusions

Pteridophytes possess bioactive compounds with potential pharmaceutical properties. Most of these compounds are similar to those in angiosperms; however, few vary in their chemical structure and activities and are exclusively found in ferns. This review covers 81 compounds specifically obtained from ferns possessing diverse biological activities, including anticancer, antidiabetic, antibacterial, antitrematodal, and hepatoprotective activities. Secondary metabolites are generally synthesized in limited quantities, but their production can be intensified by environmental stimuli to help enhance stress tolerance. Plants adapt to stress by altering their physiological, morphological, and biochemical characteristics. In ferns, contrary to angiosperms, research has been directed toward understanding the impacts of light, water, temperature, and salinity on secondary metabolite production, with only a few plant taxa and few secondary metabolites taken into consideration. Research on stimulating enhanced secondary metabolite production under the influence of abiotic stress has been focused on a few species from the families Aspleniaceae, Athyriaceae, Dryopteridaceae, Onocleaceae, Thelypteridaceae, Dennstaedtiaceae, Polypodiaceae, Pteridaceae, Salviniaceae, Cyatheaceae, and Osmundaceae. However, the literature suggests a wider group of fern families with potential bioactive compounds. Furthermore, research conducted to date in enhancing bioactive compound production in ferns through elicitors are limited to polyphenols and phenolic acids. Table 3 depicts a great diversity of compounds (apart from polyphenols and phenolic acids) that remain to be analyzed regarding the effect of elicitors on their production. Amongst these unique secondary metabolites, terpene and terpene glycoside form a major group, but studies on their in vivo scale up remain elusive. This is followed by compounds belonging to flavonoids, flavonoid glycosides, and phloroglucinol derivatives. Increased flavonoid content under the influence of abiotic stressors in species within the families Athyriaceae, Pteridaceae, Onocleaceae, Dryopteridaceae, Salviniaceae, Osmundaceae, and Dennstaedtiaceae has been discussed in this review. However, there is limited research on phloroglucinol derivatives. Moreover, research has been specifically focused on the sporophytic phase of fern life cycle. Future investigations should focus on both stages, gametophytes and sporophytes, to assess stage-specific metabolite synthesis. Furthermore, exploring the genetic regulation of these pathways using genetic engineering techniques like CRISPR Cas9 can enhance control over metabolite production. Compared to model angiosperms, the molecular regulation of secondary metabolites under abiotic stress remains poorly understood in ferns. In model plants, stress-induced accumulation of secondary metabolites is frequently associated with calcium signaling, reactive oxygen species signaling, mitogen-activated protein kinase cascades, phytohormone signaling, and transcriptional regulators controlling phenylpropanoid, flavonoid, alkaloid, and terpenoid biosynthesis. In ferns, similar regulatory mechanisms have been suggested, but only a limited number of studies have examined them using omics-based approaches. One recent integrated metabolomic and transcriptomic study on *Adiantum nelumboides* under drought, half-waterlogging, and rewatering conditions detected 864 metabolites, including flavonoids, phenolic acids, alkaloids, lignans, coumarins, and terpenoids [109]. Drought and half-waterlogging induced broad changes in primary and secondary metabolism, and these metabolic changes were accompanied by the differential expression of genes involved in related biosynthetic pathways. The same study also reported changes in the expression of genes associated with plant hormone signaling, including auxin, abscisic acid, gibberellin, and salicylic acid-related pathways, as well as increased expression of ROS-scavenging genes, such as superoxide dismutase, catalase, ascorbate peroxidase, dehydroascorbate reductase, monodehydroascorbate reductase, and glutathione reductase. These findings suggest that water-related abiotic stress responses in ferns may involve hormone-mediated and ROS-associated signaling networks linked to metabolic reprogramming. However, direct evidence connecting specific stress-signaling pathways with the biosynthesis of fern-specific metabolites, such as pterosins, pterosides, fernane-type triterpenes, phloroglucinol derivatives, or other characteristic compounds, is still lacking. Future studies should therefore integrate transcriptomics, metabolomics, proteomics, hormone profiling, and functional validation to identify the key regulators controlling stress-induced accumulation of bioactive secondary metabolites in ferns.

Acquiring sufficient quantities of these compounds for further biological testing remains a significant challenge. Most existing studies have been performed ex vitro (Table 3). Scaling up plant tissue cultures under controlled conditions with different abiotic stressors could enhance the yield of bioactive compounds. Therefore, more in vitro studies are necessary to provide a foundation for researchers to enhance the production of valuable metabolites under varying conditions. Moreover, striking a balance between upscaling secondary metabolite production and avoiding the potentially damaging effects of abiotic stress on culture growth is a crucial challenge in this elicitation approach. Researchers must carefully optimize the stress dosage/adopt a combinatorial strategy to upscale secondary metabolite production. Abiotic stress-induced enhancement of secondary metabolite production should be interpreted in the context of the growth–defense trade-off. Under stress conditions, plants frequently redirect carbon skeletons, ATP, NADPH, and other metabolic resources from primary metabolism, cell expansion, and biomass formation toward the biosynthesis of protective secondary metabolites. In ferns, as in other plant groups, this shift may increase the concentration of phenolic compounds, flavonoids, carotenoids, terpenoids, or other defense-related metabolites. However, the same stress conditions may also reduce photosynthetic efficiency, nutrient uptake, water status, and growth rate. Consequently, an apparent increase in metabolite concentration does not necessarily translate into a higher total yield of bioactive compounds if biomass production is strongly reduced. Therefore, the optimization of abiotic stress as an elicitation strategy should consider both metabolite concentration and total metabolite yield per plant or per cultivation unit. From a practical perspective, moderate, transient, or stage-specific stress treatments may be more beneficial than severe or prolonged stress because they can stimulate defense-related metabolic pathways while limiting growth inhibition. Future studies on ferns should therefore determine stress thresholds, exposure duration, recovery periods, and developmental stages that maximize the accumulation of target metabolites without causing unacceptable biomass losses. Such an approach would allow a more rational balance between growth and defense and could support the development of controlled cultivation systems for producing fern-derived bioactive compounds.

## Figures and Tables

**Table 3:** Influence of abiotic stress on secondary metabolite production in ferns.

Sr. No.	Family	Species	Growth Conditions	Stress Factor	Metabolite Production Under Stress	References
Light						
1	Athyriaceae	*Athyrium nipponicum*	Outdoor pot experiment	Full sunlight (3-month mean 551.9–890.1 µmol m^−2^ s^−1^)	(-) Flavonoids and (-) total polyphenols	[97]
2	Athyriaceae	*Athyrium multidentatum* (Doll.) Ching	Outdoor pot experiment	35% full sunlight	↑ Flavonoids, and ↑ total phenols	[98]
3	Athyriaceae			4% full sunlight	↓ Flavonoids, and ↓ total phenols	[98]
4	Cyatheaceae	*Cyathea delgadii* Sternb.	In vitro	Fluorescent lamps	↑ Protocatechuic acid, ↑ cis-5-O-caffeoylquinic acid, ↑ caffeic acid, ↑ quercitrin, and ↑ naringenin 7-O-glucoside	[99]
5	Cyatheaceae		In vitro	100% blue LED light (430 nm)	↑ Rutin, ↑ isoquercetin, ↑ nicotiflorin, and ↑ astragalin	[99]
6	Cyatheaceae		In vitro	Combination of red and blue LED light (70%/30%)	↑ trans-5-O-caffeoylquinic acid	[99]
7	Dennstaedtiaceae	*Pteridium aquilinum* (L.) Kuhn var. *latiusculum* (Desy.) Underw. ex Heller	Outdoor pot experiment	13% full sunlight	↑ Total phenols	[98]
8	Dennstaedtiaceae			4% full sunlight	↑ Flavonoids	[98]
9	Dicksoniaceae	*Dicksonia antarctica*	Glasshouse	900 mmol photons m^−2^ s ^−1^ (high irradiance)	↑ Violaxanthin, ↑ antheraxanthin, ↑ zeaxanthin, ↑ neoxanthin, ↑ lutein, and ↑ α-tocopherol	[100]
10	Dicksoniaceae	*Dicksonia antarctica* Labill.,	Climate chamber	130 to 500 µmol m^−2^ s^−1^	↑ Isoprene	[101]
11	Dryopteridaceae	*Dryopteris erythrosora*	Outdoor pot experiment	Full sunlight (3-month mean 551.9–890.1 µmol m^−2^ s^−1^)	↑ Flavonoids and (-) total polyphenols	[97]
12	Dryopteridaceae	*Polystichum setiferum*	Greenhouse	(100% full sunlight) ~525 μmol m^−2^ s^−1^ PPFD	↓ Carotenoids, ↓ polyphenols, and ↓ flavonoids	[102]
13	Onocleaceae	*Matteuccia struthiopteris* (L.) Todar. *	Outdoor pot experiment	35% full sunlight	↑ Flavonoids and ↑ total phenols	[98]
14	Onocleaceae			4% full sunlight	↓ Total phenols	[98]
15	Osmundaceae	*Osmunda cinnamomea* (L.) var. *asiatica* Fernald	Outdoor pot experiment	35% full sunlight	↑ Flavonoids and ↑ total phenols	[98]
16	Osmundaceae			8% full sunlight	↓ Flavonoids and ↓ total phenols	[98]
17	Pteridaceae	*Acrostichum danaeifolium*	In vitro	Ultraviolet B radiation (UV-B)	↓ Lutein, ↓ zeaxanthin, ↑ trans-β-carotene, and ↑ total polyphenols	[103]
18	Salviniaceae	*Azolla microphylla* Kaulf.	Greenhouse	Ultraviolet C radiation (UV-C)	↑ Anthocyanin, ↑ flavonoids, and ↓ carotenoids	[104]
19	Thelypteridaceae	*Thelypteris kunthii* (Desv,) Morto	Climate chamber	130 to 500 µmol m^−2^ s^−1^	↑ Isoprene	[101]
20	Thelypteridaceae	*Thelypteris decursive-pinnata* (Van Hall) Ching	Climate chamber	130 to 500 µmol m^−2^ s^−1^	↑ Isoprene	[101]
Temperature						
21	Dicksoniaceae	*Dicksonia antarctica* Labill.,	Climate chamber	35 and 39 °C	↑ Isoprene	[101]
22	Dicksoniaceae	*Dicksonia antarctica*	Glasshouse	35 °C	(-) Zeaxanthin, (-) α-tocopherol, (-) violaxanthin, (-) antheraxanthin, (-) neoxanthin, (-) lutein, and ↑ β-carotene	[100]
23	Thelypteridaceae	*Thelypteris kunthii* (Desv,) Morto	Climate chamber	35 and 39 °C	↑ Isoprene	[101]
24	Thelypteridaceae	*Thelypteris decursive-pinnata* (Van Hall) Ching	Climate chamber	35 and 39 °C	↑ Isoprene	[101]
Salt						
25	Aspleniaceae	*Asplenium viride* Britton	In vitro	100 mM NaCl	↑ Total phenolic content	[105]
26	Aspleniaceae	*Ceterach officinarum* DC	In vitro	100 mM NaCl	↑ Total phenolic content	[105]
27	Aspleniaceae	*Phyllitis scolopendrium* (L.) Newman	In vitro	100 mM NaCl	↑ Total phenolic content	[105]
28	Athyriaceae	*Athyrium nipponicum*	Outdoor pot experiment	100 mmol dm^−3^ NaCl	↑ Flavonoids	[97]
29	Athyriaceae	*Athyrium nipponicum*	Unheated plastic tunnel with shades installed	100 mmol dm^−3^ CaCl_2_	↑ carotenoids, ↑ total polyphenols, and ↑ total flavonoids	[106]
30	Dryopteridaceae	*Dryopteris erythrosora*	Outdoor pot experiment	101 mmol dm^−3^ NaCl	↑ Flavonoids	[97]
Drought						
31	Athyriaceae	*Athyrium multidentatum* (Doll.) Ching	Outdoor pot experiment	5% soil moisture	↑ Total phenolics	[107]
32	Athyriaceae		Outdoor pot experiment	5% soil moisture	↑ Proanthocyanidin content	[107]
33	Athyriaceae	*Athyrium nipponicum*	Unheated plastic tunnel with shades installed	−400 hPa substrate water potential	↑ Carotenoids, ↓ total polyphenols, and ↓ total flavonoids	[106]
34	Polypodiaceae	*Polypodium vulgare*	Grown in wild	Dehydrated in mannitol	↑ Total phenols	[108]
35	Pteridaceae	*Adiantum nelumboides*	Outdoor pot experiment	30% soil moisture	↑ Phenolic acids, ↑ alkaloids, ↑ flavonoids, and ↓ terpenoids	[109]
36	Onocleaceae	*Matteuccia struthiopteris* (L.) Todar.	Outdoor pot experiment	17% soil moisture	↑ Flavonoids	[107]
37	Onocleaceae		Outdoor pot experiment	2% soil moisture	↑ Total phenolics	[107]
38	Onocleaceae		Outdoor pot experiment	5% soil moisture	↑ Proanthocyanidin content	[107]
Nutrient Composition						
39	Athyriaceae	*Diplazium maximum*	In vitro	3% sucrose	↑ Proline, ↑ superoxide dismutase, ↑ ascorbate peroxidase, and ↑ glutathione reductase	[110]

↑ increase; ↓ decrease; (-) no change. * The authors of the Latin names are cited as they were written in the article.

## Data Availability

No new data were created or analyzed in this study. Data sharing is not applicable to this study.
